# Bempedoic Acid: Lipid Lowering for Cardiovascular Disease Prevention

**DOI:** 10.17925/HI.2023.17.2.1

**Published:** 2023-11-01

**Authors:** Michael Albosta, Jelani K Grant, Erin D Michos

**Affiliations:** 1. Internal Medicine Department, University of Miami Miller School of Medicine/Jackson Memorial Hospital, Miami, FL, USA; 2. Ciccarone Center for the Prevention of Cardiovascular Disease, Division of Cardiology, Johns Hopkins University School of Medicine, Baltimore, MD, USA

**Keywords:** Atherosclerotic cardiovascular disease, bempedoic acid, cardiovascular disease prevention, clear outcomes trial, lipids, lipid-lowering, low-density lipoprotein cholesterol

## Abstract

The management of low-density lipoprotein cholesterol (LDL-C) levels is a central strategy for the prevention of atherosclerotic cardiovascular disease. Current United States (2018 American Heart Association/American College of Cardiology/Multisociety) and European (2019 European Society of Cardiology/European Atherosclerosis Society) guidelines endorse statin therapy as the first-line therapy for pharmacologic LDL-C lowering. However, in clinical practice up to 30% of patients report partial or complete intolerance to statin therapy. While the nocebo effect with statins is well described, perceived statin intolerance prevents many patients from achieving LDL-C thresholds associated with clinical benefit. Bempedoic acid is a novel, oral, non-statin lipid-l owering therapy that works by inhibiting adenosine triphosphate-citrate lyase, an enzymatic reaction upstream of 3-hydroxy-3-methylglutaryl coenzyme A reductase in the hepatic cholesterol synthesis pathway. Bempedoic acid confers reduction in LDL-C of ~18% on background statin therapy,~21% in patients with statin intolerance, and ~38% when given in fixed-dose combination with ezetimibe. The CLEAR Outcomes trial, which enrolled high-risk primary and secondary prevention patients with reported statin intolerance and LDL-C levels ≥100 mg/dL, showed that bempedoic acid compared with placebo reduced 4-component major adverse cardiovascular events (MACE) by 13% (hazard ratio 0.87, 95% confidence interval 0.79–0.96). Bempedoic acid also reduced 3-component MACE by 15%, myocardial infarction by 23% and coronary revascularization by 19%. The benefit was even greater in the primary prevention cohort (hazard ratio 0.70, 95% confidence interval 0.55–0.89) for 4-component MACE. Bempedoic acid was associated with increases in uric acid levels and cholelithiasis, but numerically fewer events of myalgia and new-onset diabetes. These findings confirm that bempedoic acid is an effective approach to reduce cardiovascular outcomes in high-risk patients with statin intolerance who require further reduction in LDL-C.

Atherosclerotic cardiovascular disease (ASCVD) remains the leading cause of mortality both in the US and worldwide.^1^ There is substantial evidence from genetic, observational and interventional studies that low-density lipoprotein cholesterol (LDL-C) is causally related to the development of ASCVD in a dose-dependent fashion.^2,3^ Statins have been shown to substantially lower LDL-C and form the cornerstone of lipid-l owering therapies endorsed by the American College of Cardiology (ACC)/American Heart Association, as well as the European Society of Cardiology/European Atherosclerosis Society guidelines, as first-l ine agents for the management of hyperlipidaemias.^4,5^ Despite extensive evidence of statin efficacy and safety,^6,7^ statin intolerance (or perceived intolerance) is common in clinical practice and is cited as a frequent reason for discontinuation or non-adherence with statin therapy.^8–10^ This is concerning, as suboptimal statin adherence is associated with an increased risk for major adverse cardiovascular events (MACE).^11–13^

While the pooled prevalence of statin intolerance according to definitions by the National Lipid Association (NLA), European Atherosclerosis Society, and International Lipid Expert Panel is approximately 9.1%, there is significant discrepancy between the rates seen in randomized controlled trials of 5–7% and those observed in cohort studies (up to 30%).^14^ Despite data from the SAMSON trial ( ClinicalTrials. gov identifier: NCT02668016) suggesting that the majority of adverse symptoms related to statin therapy are due to the nocebo effect,^15^ adherence to statin therapy in clinical practice remains challenging. Therefore, there is a role for effective non-statin therapy for LDL-C lowering in the management of ASCVD risk.^16^

Bempedoic acid is a novel lipid-l owering therapy that works by inhibiting adenosine triphosphate (ATP)-citrate lyase, an enzyme upstream of 3-hydroxy-3-methylglutaryl coenzyme A (HMG-CoA) reductase (the target of statins) in the cholesterol synthesis pathway.^17^ By being a prodrug that is only activated in hepatic cells, bempedoic acid is not associated with the adverse muscle effects that have been reported with statin therapy.^18^ As such, the recent Cholesterol lowering via bempedoic acid, an ATP citrate lyase-i nhibiting regimen (CLEAR) Outcomes trial ( ClinicalTrials. gov identifier: NCT02993406) investigated the use of bempedoic acid in high-risk primary and secondary prevention patients who were either unable or unwilling to take statin therapy due to intolerance.^18^ The objective of this review is to discuss the safety and efficacy of bempedoic acid, outline the cardiovascular outcomes trial data, and provide considerations for the use of bempedoic acid in contemporary practice.

**Figure 1:**
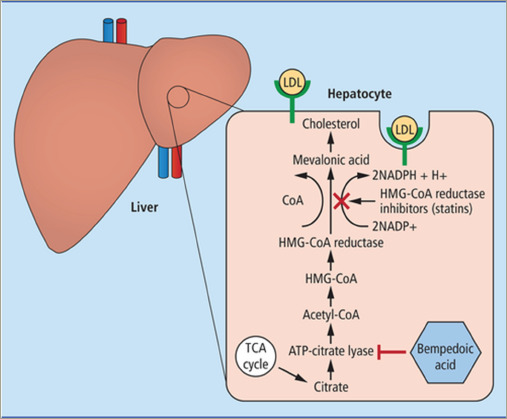
Mechanism of action of bempedoic acid

*Overview of the mechanism of bempedoic acid as an inhibitor of ATP-citrate lyase in the cholesterol synthesis pathway. The red line represents inhibition of ATP-citrate lyase. The red X represents inhibition of HMG-CoA reductase, the mechanism of action of statins.*

*ATP = adenosine triphosphate; CoA = coenzyme A; HMG-CoA = 3-hydroxy-3-methylglutaryl coenzyme A; LDL = low-density lipoprotein; NADPH = nicotinamide adenine dinucleotide phosphate; TCA = tricarboxylic acid*

*Reproduced with permission and without amendment from Oroko et al.^19^* (http://creativecommons.org/licenses/by-nc/4.0).

## Mechanism of action

Cholesterol synthesis in the liver occurs via a complex, heavily regulated pathway involving several enzymatic reactions, starting with acetyl-CoA and ending with cholesterol. Bempedoic acid works by inhibiting ATP-citrate lyase (*Figure 1*),^19^ and thus works upstream of the enzyme inhibited by statin therapy in the cholesterol synthesis pathway.^20,21^ This inhibition leads to a decreased production of intra-hepatic cholesterol, with the subsequent upregulation of hepatic LDL-receptors.^20^ This upregulation of LDL-receptors leads to an increased clearance of LDL particles from the bloodstream and therefore, lowers LDL-C levels.

**Figure 2:**
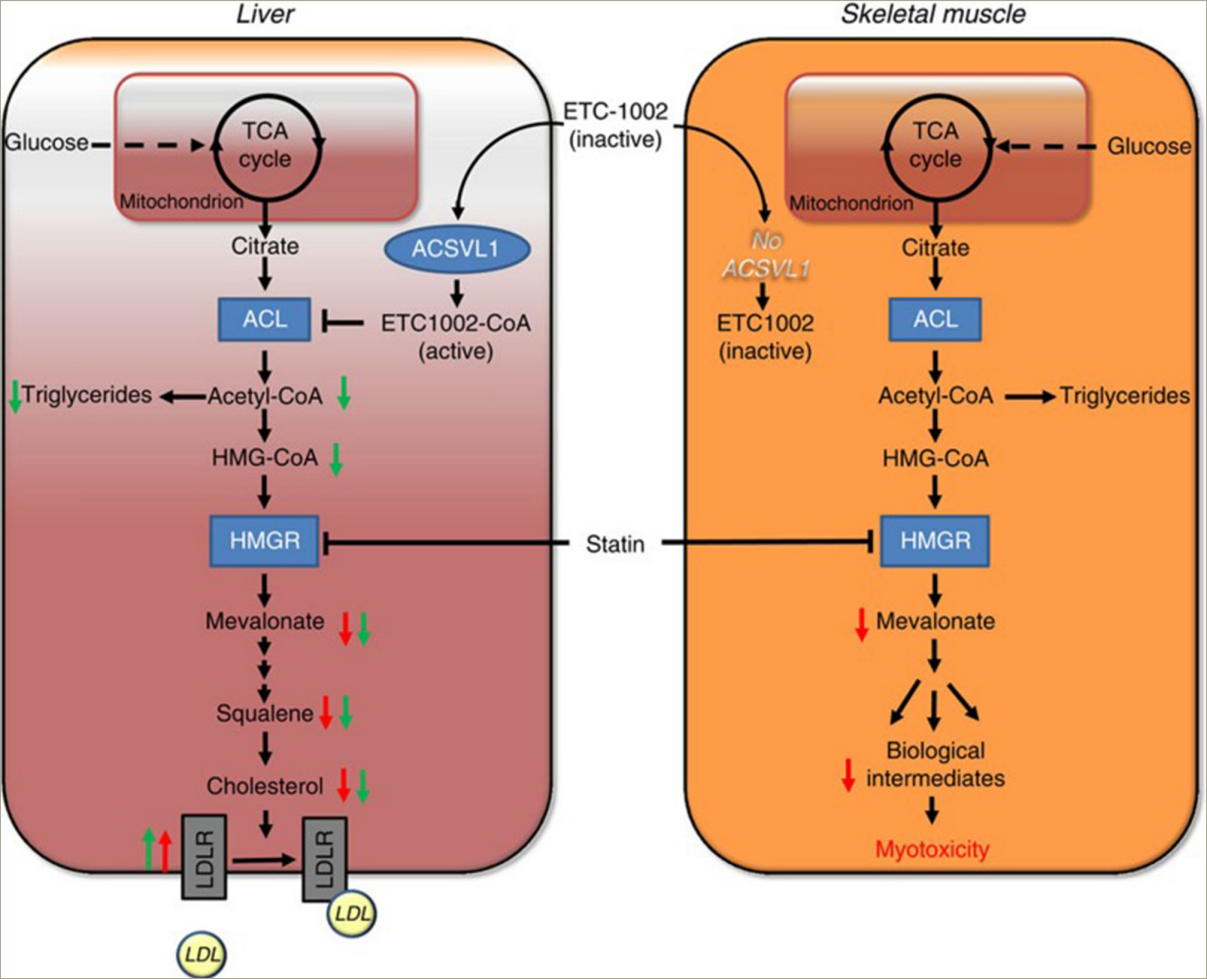
Overview of the targeted uptake of bempedoic acid (ETC-1002) into hepatocytes

Bempedoic acid is administered as a prodrug that requires activation by a liver-specific enzyme called very long-chain acyl-CoA synthetase-1 (ACSVL1).^20^ This enzyme is present in hepatocytes and not present in skeletal muscle cells, therefore allowing for targeted uptake by the liver (*Figure 2*).^20^ This preferential uptake by hepatocytes provides a potential explanation for the absence of the muscle-associated symptoms that have been observed with statin therapy.

## Dosing and route of administration

Bempedoic acid is administered as a simple, once-daily, oral medication at a dose of 180 mg per day or as a fixed-dose combination pill with ezetimibe (bempedoic acid/ezetimibe) administered orally at a dose of 180 mg/10 mg once daily.^22,23^ Ezetimibe inhibits the Niemann-Pick C1-Like 1 sterol transporter in the small intestine, thus decreasing intestinal absorption of cholesterol.^24^ Given together, these medications act synergistically to reduce both cholesterol synthesis and absorption, leading to a greater degree of LDL-C lowering than is expected from either medication alone, and similar to that seen in patients taking at least a moderate-i ntensity statin.^25,26^ For this purpose, bempedoic acid, either alone or in combination with ezetimibe, is a reasonable alternative for patients with statin-associated muscle symptoms (SAMS) who require additional LDL-C lowering. Bempedoic acid can also be used on a background of statin therapy if further LDL-C lowering is needed.

## Clinical efficacy

The efficacy of bempedoic acid for LDL-C lowering was first demonstrated in phase II clinical trials.^27–29^ This was followed by the CLEAR trial series, a group of phase III clinical trials that sought to assess the safety and efficacy of bempedoic acid (*Table 1*).^25,30–33^ CLEAR Harmony and CLEAR Wisdom investigated bempedoic acid on top of background statin therapy, while CLEAR Tranquility and CLEAR Serenity included patients with statin intolerance.

CLEAR Harmony evaluated the safety and efficacy of bempedoic acid in patients with ASCVD, heterozygous familial hypercholesterolaemia (FH), or both over a period of 52 weeks.^30^ Prior to CLEAR Harmony,^30^ data regarding bempedoic acid were limited to short-term studies with a median follow-up period of 12 weeks. CLEAR Harmony included 2,230 patients randomized to bempedoic acid or placebo on a background of maximally tolerated statin therapy ± additional lipid-l owering therapy who remained with an LDL-C ≥70 mg/dL. Bempedoic acid reduced LDL-C at Week 12 by 18.1%, Week 24 by 16.1% and Week 52 by 13.6%. At Week 12, bempedoic acid conferred reductions in non-high-density lipoprotein cholesterol (non-HDL-C), apolipoprotein B (apoB) and high-sensitivity C-reactive protein (hsCRP) of 13.3%, 11.9%, and 21.5%, respectively; these reductions were sustained at Week 52: 10.5% (non-HDL-C), 9.0% (apoB) and 16.2% (hsCRP). Overall, bempedoic acid was found to effectively decrease LDL-C, non-HDL-C, apoB and hsCRP in a sustained manner in patients on a background of maximally tolerated statin therapy.

*ACL = adenosine triphosphate-citrate lyase; ACSVL1 = very long-chain acyl-coenzyme A synthetase; HMG-CoA = 3-hydroxy-3-methylglutaryl coenzyme A; HMGR = 3-hydroxy-3-methylglutaryl coenzyme A reductase; LDL = low-density lipoprotein; LDLR = low-density lipoprotein receptor; TCA = tricarboxylic acid*

*Reproduced with permission and without amendment from Pinkosky et al.*^20^ (http://creativecommons.org/licenses/by/4.0/)

CLEAR Wisdom evaluated the efficacy of bempedoic acid in 779 high-risk patients with a history of ASCVD, heterozygous FH or both, and a LDL-C level ≥70 mg/dL on stable, maximally tolerated lipid-l owering therapy.^31^ However, unlike CLEAR Harmony, patients were not excluded if they were unable to tolerate any dose of statin therapy, and therefore patients were included in the trial on a statin or alternative background lipid-l owering therapy. Bempedoic acid significantly lowered levels of LDL-C at a placebo-corrected least-squares mean difference of 17.4% (95% confidence interval [CI] 13.9–21.0%) at Week 12, and by 14.8% (95% CI 10.0–19.5%) at Week 24. Similar to prior trials, there were significant reductions in non-HDL-C, apoB, and hsCRP (*Table 1*).

CLEAR Tranquility was a phase III, multicentre, randomized controlled trial evaluating the safety and efficacy of bempedoic acid with ezetimibe in 269 patients with LDL-C ≥100 mg/dL and either had a history of statin intolerance or were on low-dose statin therapy.^25^ Bempedoic acid at a dose of 180 mg daily led to a 28.5% (95% CI 22.5–34.4%) reduction in LDL-C over the 12 week trial period. In addition, non-HDL-C and apoB were decreased by 23.6% and 19.3%, respectively, and hsCRP was reduced by a median of 33.0%. Overall, the investigators concluded that bempedoic acid was effective in reducing levels of atherogenic lipoproteins, including in patients with intolerance to statins.

CLEAR Serenity was the final phase III trial in the CLEAR series evaluating the safety and efficacy of bempedoic acid.^32^ CLEAR Serenity evaluated 345 patients with a history of statin intolerance and an LDL-C level of ≥130 mg/dL (≥100 mg/dL if heterozygous FH), who required additional lipid-l owering for either primary or secondary prevention. At Week 24, patients receiving bempedoic acid had a 18.9% (95% CI 14.9–23.0%) reduction in LDL-C from baseline. Those receiving bempedoic acid demonstrated significant reductions in total cholesterol, non-HDL-C, apoB and hsCRP.

Recently, the CLEAR Harmony open-l abel extension study was published, providing long-term data (up to 130 weeks) for those initially enrolled in the CLEAR Harmony trial.^33^ Of the initial 2,230 patients in CLEAR Harmony trial, 1,462 were enrolled in the extension study. Patients who received bempedoic acid for a total of 130 weeks were found to have sustained LDL-C reductions of 14.2 ± 0.9%. In addition, the mean percent change for additional lipid parameters included a 11% reduction in non-HDL-C, 7% reduction in apoB, 10% reduction in total cholesterol and a median 17% reduction from baseline in hsCRP.^33^

While the majority of the phase III trials evaluated bempedoic acid either as monotherapy or on background statin therapy, a recently published phase III trial evaluated the efficacy of a fixed-dose combination of bempedoic acid (180 mg) and ezetimibe (10 mg) in 382 high-risk patients (defined as having a history of ASCVD, heterozygous FH or multiple ASCVD risk factors) on a background of maximally tolerated statin therapy.^34^ The study was carried out for a total of 12 weeks, and patients were randomized to receive either bempedoic acid/ezetimibe, bempedoic acid monotherapy, ezetimibe monotherapy or placebo. Patients receiving the combination of bempedoic acid/ezetimibe observed a 38% placebo-corrected reduction in LDL-C, compared with a 2% increase in LDL-C in placebo arm; whereas, ezetimibe and bempedoic acid monotherapies reduced LDL-C by 23% and 17%, respectively.^34^ In addition, 33.7% of patients in the bempedoic acid/ezetimibe combination group had >50% reductions in LDL-C. Bempedoic acid/ezetimibe yielded a significant 35.1% reduction in hsCRP, compared with a 31.9% reduction in the bempedoic acid monotherapy group and an 8.2% reduction in patients on ezetimibe monotherapy. Further, the bempedoic acid/ezetimibe combination yielded greater reductions in total cholesterol (26.4%), non-HDL-C (31.9%) and apoB (24.6%).

**Table 1:** Summary of the phase III cholesterol lowering via bempedoic acid, an ATP-citrate lyase inhibiting regimen (CLEAR) trial series^25,30–32^

	CLEAR Tranquility (ClinicalTrials.gov identifier: NCT03001076)^25^	CLEAR Harmony (ClinicalTrials.gov identifier: NCT02666664)^30^	CLEAR Wisdom (ClinicalTrials.gov identifier: NCT02991118)^31^	CLEAR Serenity (ClinicalTrials.gov identifier: NCT02988115)^32^
**Patient population**	Patients with statin intolerance or those on no more than low-dose statin therapy with LDL-C ≥100 mg/dL	Adults with ASCVD, heterozygous (FH) or both, and LDL-C ≥70 mg/ dL while on maximally tolerated statin therapy	Adults with ASCVD, heterozygous (FH) or both, and LDL-C ≥70 mg/dL while on maximally tolerated lipid-l owering therapy	Adults with LDL ≥130 mg/dL (≥100 if heterozygous FH) who require additional lipid-l owering for either primary/secondary prevention with a history of statin intolerance
**Number of participants**	269	2,230	779	345
**Baseline LDL-C (bempedoic acid versus placebo)**	129.8 mg/dL (bempedoic acid), 123.0 mg/dL (placebo)	103.6 ± 29.1 mg/dL (bempedoic acid), 102.3 ± 30.0 mg/dL (placebo)	119.4 mg/dL (bempedoic acid), 122.4 mg/dL (placebo)	158.5 ± 40.4 mg/dL (bempedoic acid), 155.6 ± 38.8 mg/dL (placebo)
**Percent change in LDL-C**	Placebo-corrected percent change at 12 weeks:-28.5%	Mean difference: Week 12: -18.1% Week 52: -13.6%	Placebo-corrected mean difference: Week 12: -17.4% Week 24: -14.8%	Placebo-corrected percent change: Week 12: -21.4% Week 24: -18.9%
**Other notable findings**	Non-HDL-C decreased by 23.6% ApoB decreased by 19.3% Total cholesterol decreased by 18.0% TG decreased by 1.4% hsCRP decreased by a median of 33.0%	*Non-HDL-C* Week 12: -13.4% Week 52: -10.5% *Total cholesterol* Week 12: -11.1% Week 52: -9.2% *ApoB* Week 12: -11.9% Week 52: -9.0% *hsCRP* Week 12: -25.0% Week 52: -16.2% *Triglycerides: no significant change*	*Non-HDL-C* Week 12: -13.0% Week 52: -9.9% *Total cholesterol* Week 12: -11.2% Week 52: -8.4% *ApoB* Week 12: -13.0% Week 52: -9.6% *hsCRP* Week 12: -8.7% Week 52: -7.6% *Triglycerides*: no significant change	*Non-HDL-C* Week 12: -18.6% Week 24: -17.1% *Total cholesterol* Week 12: -15.5% Week 24: -14.5% *ApoB* Week 12: -15.3% Week 24: -15.5% *hsCRP* Week 12: -28.1% Week 24: -27.1% *Triglycerides:* no significant change
**Adverse effects (bempedoic acid versus placebo**)	*Increased serum uric acid: 7.7%* versus 2.3% *Headache:* 4.4% versus 3.4% *Elevated liver function test:* 3.9% versus 0% *Nausea:* 2.8% versus 0% *Sinusitis:* 2.8% versus 0% *Nasopharyngitis:* 2.2% versus 1.1% *New-onset diabetes:* 1.1% versus 2.3% *Muscle-related adverse events:* 3.3% versus 3.4%	*Nasopharyngitis:* 9.8% versus 11.7% *Muscular disorders (overall):* 13.1% versus 10.1% *Myalgia:* 6.0% versus 6.1% *Muscle spasm:* 4.2% versus 2.7% *Pain in extremity:* 3.4% versus 2.2% *Gout:* 1.2% versus 0.3% *New-onset or worsening diabetes:* 3.3% versus 5.4%	*Nasopharyngitis:* 5.2% versus 5.1% *Urinary tract infection:* 5.0% versus 1.9% *Elevated uric acid:* 4.2% versus 1.9% *Gout:* 2.1% versus 0.8% *New-onset or worsening diabetes:* 6.9% versus 7.4% *Elevated liver function test:* 1.1% versus 0.8% *Myalgias:* 2.9% versus 3.1% *Muscle spasm:* 2.1% versus 1.2% *Muscle weakness:* 0.4% versus 0.4%	*Arthralgia:* 6.0% versus 4.5% *Hypertension:* 4.3% versus 1.8% *Muscle-related adverse events:* 12.8% versus 16.2% *Pain in extremity:* 5.6% versus 3.6% *Myalgia:* 4.7% versus 7.2% *Muscular weakness:* 0.4% versus 1.8% *New-onset or worsening diabetes:* 2.1% versus 4.5% *Gout:* 1.7% versus 0.9%

*ApoB = apolipoprotein B; ASCVD = atherosclerotic cardiovascular disease; CLEAR = cholesterol lowering via bempedoic acid, an adenosine triphosphate citrate lyase-inhibiting regimen; FH = familial hypercholesterolaemia; HDL-C = high-density lipoprotein cholesterol; hsCRP = high-sensitivity C-reactive protein; LDL-C = low-density lipoprotein cholesterol; TG = triglycerides.*

## Safety data

Prior to the publication of CLEAR Outcomes data, the safety of bempedoic acid was evaluated in each of the phase III CLEAR trials.^35^ Overall, safety data were collected from a total of 3,621 patients. The overall exposure-adjusted rate of treatment-emergent adverse events was 87.1/100 person-years in those receiving bempedoic acid compared with 82.9/100 person-years in those receiving placebo.^35^ The most common adverse events were nasopharyngitis, myalgia, urinary tract infections and arthralgia. Each of the clinical trials sought to identify adverse events of special interest based on the safety outcomes of prior phase I or II trials for bempedoic acid, and known adverse events from statins or other lipid-l owering therapies. The most prominent of these adverse events of special interest included elevated hepatic enzymes, muscle symptoms, and elevations in serum uric acid/gout.^35^ Overall, the incidence of hepatic enzyme elevation occurred in 3.3/100 person-y ears compared with 1.4/100 person-years in patients taking bempedoic acid and placebo, respectively. Of note, transaminase levels returned to <3 times the upper limit of normal in all patients, regardless of whether bempedoic acid was discontinued or not.

Muscle-related symptoms were found to occur at a rate of 15.4/100 person-years in patients taking bempedoic acid, compared with 11.9/100 person-years in those taking placebo.^35^ The incidence of myalgias and muscular weakness were similar between groups. Of note, there were no cases of myopathy or rhabdomyolysis reported.

**Figure 3:**
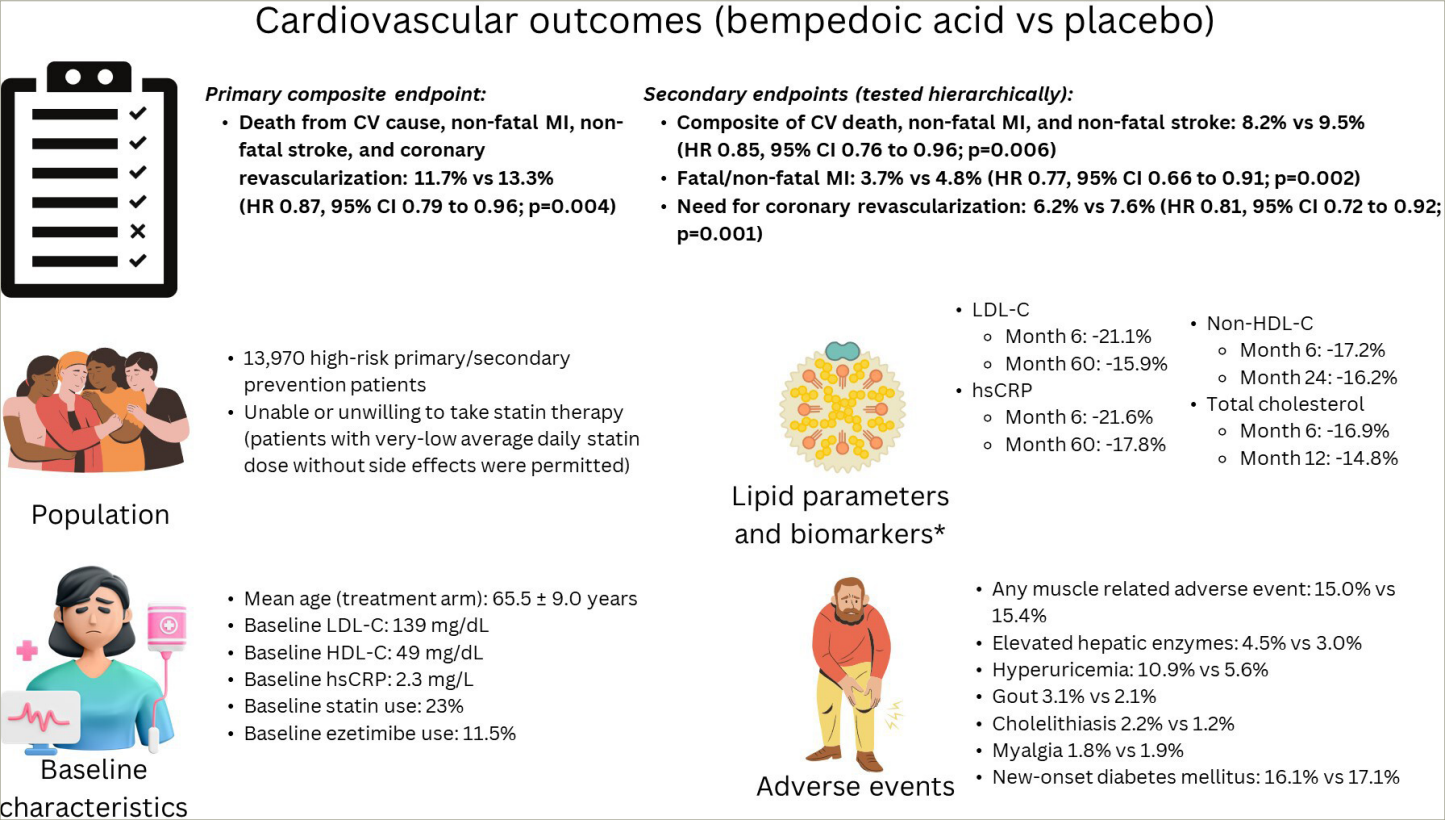
Overview of CLEAR Outcomes cardiovascluar outcomes trial^18^

*Overview of the results of the CLEAR Outcomes trial evaluating the impact of bempedoic acid compared with placebo in statin-intolerant patients.*

*Mean percent change from baseline.

*CI = confidence interval; CV = cardiovascular; HDL-C = high-density lipoprotein cholesterol; HR = hazard ratio; hsCRP = high sensitivity C-reactive protein; LDL-C = low-density lipoprotein cholesterol; MI = myocardioal infarction*

Patients taking bempedoic acid had an increased incidence of elevated serum uric acid levels (an approximate increase of +0.82 mg/dL) that were sustained and reversible after stopping treatment.^35^ Further, the incidence of gout was greater in patients taking bempedoic acid compared with placebo (1.6 versus 0.5/100 person-years). Of note, the increased incidence of gout was greater in patients with a documented history of gout compared with those who did not.

Finally, rates of new-onset diabetes were lower in the bempedoic acid group than those receiving placebo (4.7 versus 6.4/100 person years), which is in contrast to what has been reported with statin therapy.^35,36^ New-onset diabetes was defined as a fasting glucose ≥126 mg/dL or haemoglobin A1C ≥6.5%. ATP-citrate lyase is involved in pathways of both lipid and carbohydrate metabolism, and as such there may be mechanistic plausibility of how ATP-citrate lyase inhibition may confer improved glycaemic variables; however, additional mechanistic studies are required.^37^

## Cardiovascular outcomes data

Published in March 2023, CLEAR Outcomes was a double-blind, randomized, placebo-controlled trial that sought to evaluate cardiovascular (CV) outcomes in high-risk primary and secondary prevention patients who were either unable or unwilling to take statin therapy due to intolerance (*Figure 3*), and remained with an LDL-C level of ≥100 mg/dL.^18^ It was required that participants signed a written statement acknowledging their awareness that statins reduce CV events, including death, and many patients with statin intolerance can receive a different statin or dose, though despite that they were still unable to take them. A written statement of confirmation was also required by the enrolling study investigators. Patients who were able to tolerate a very-l ow-dose statin therapy (i.e. daily doses of rosuvastatin <5 mg, atorvastatin <10 mg, simvastatin <10 mg, lovastatin <20 mg, pravastatin <40 mg, fluvastatin <40 mg or pitavastatin <2 mg) were allowed to be included provided that their dose was stable, and background statin therapy was used in 23% of enrolled participants at baseline.

Patients were randomized to either bempedoic acid (180 mg/day) or placebo and followed for a median of 40.6 months. The primary endpoint was a composite of death from a CV cause, non-fatal myocardial infarction (MI), non-fatal stroke and coronary revascularization. Secondary endpoints were tested hierarchically and included the following: a composite of CV death, non-fatal MI, and non-fatal stroke, followed by fatal/non-fatal MI; the need for coronary revascularization; fatal/non-fatal stroke; CV death; and death from any cause.^18^

Patients randomized to bempedoic acid had a 21.1% reduction in LDL-C at 6 months. At Month 60, reductions in LDL-C were sustained with a 15.9% reduction in patients taking bempedoic acid compared with placebo. The primary endpoint of 4-component MACE occurred in 11.7% of patients taking bempedoic acid compared with 13.3% with placebo, representing an overall 13% relative risk reduction (hazard ratio [HR] 0.87; 95% CI 0.79–0.96; p=0.004). Additionally, there was a 15% reduction in the 3-component MACE composite of CV death, non-fatal MI, and non-fatal stroke, a 23% reduction in fatal/non-fatal MI, and 19% reduction in need for coronary revascularization. Similar to the earlier phase III studies, rates of elevated hepatic enzymes (4.5% versus 3.0%), hyperuricaemia (10.9% versus 5.6%), gout (3.1% versus 2.1%) and cholelithiasis (2.2% versus 1.2%) were greater in patients taking bempedoic acid. The incidence of myalgias (5.6% versus 6.8%) and new-onset diabetes (16.1% versus 17.1%) were numerically lower in the bempedoic acid group compared with placebo. Tendon rupture was not statistically significant between groups (1.2% versus 0.9%).

Notably, 30% of participants were high-risk primary prevention patients, and the efficacy of bempedoic acid in reducing 4-component MACE was greater in the primary prevention cohort (HR 0.68, 95% CI 0.53–0.87) than the secondary prevention cohort (HR 0.91, 95% CI 0.82–1.01), p-value for interaction of 0.03, which may be explained by an increase of additional use of other therapies (such as statins, proprotein convertase subtilisin/ kexin 9 [PCSK9] inhibitors) outside of the trial in the secondary prevention cohort. A *post hoc* analysis of the primary prevention cohort (66% were patients with diabetes) confirmed a benefit of bempedoic acid in these patients, with a 30% relative-risk reduction and 2.3% absolute reduction in 4-component MACE (HR 0.70, 95% CI 0.55–0.89; p=0.002).^38^ In addition, there were significant reductions in 3-component MACE, MI, CV death and all-cause mortality with bempedoic acid in the primary prevention cohort. These findings reaffirm the potential important utility of using bempedoic acid in the primary prevention setting in patients who are unable to take an adequate dosing of statin therapy needed to achieve desired risk-based LDL-C thresholds.

In contrast to many prior CV outcome trials that have historically underenrolled females relative to their disease burden in the population, nearly half (48%) of the participants in CLEAR Outcomes were females.^39–41^ There was no interaction for the efficacy of bempedoic acid by sex (p-value for interaction 0.89), meaning that this therapy was similarly beneficial in both males and females. Furthermore, females are more likely to have statin-a ssociated symptoms and more likely to discontinue statin therapy for these reasons.^42,43^ Thus, bempedoic acid may be a particularly attractive approach to address LDL-C lowering in this population.

Overall, the findings of the CLEAR Outcomes study demonstrate that bempedoic acid is well tolerated and effective in improving CV outcomes in patients with statin intolerance who require further LDL-C lowering. The reduction in MACE with bempedoic acid was exactly in line with what would be anticipated by the degree of LDL-C lowering. Per the Cholesterol Treatment Trialists (CTT) estimation (extrapolated from pooled statin trials), a 0.67 mmol/L (26 mg/dL) reduction in LDL-C would have an expected HR for 3-component MACE of 0.85, and that was observed in the CLEAR Outcomes trial.^18^ The definition of MACE in the CLEAR Outcomes trial was nearly identical to that used by the CTT’s meta-analysis for major vascular events, with the exception that the CTT’s definition of CV death was subdivided into those definitely due to coronary disease, and those that were due to sudden death, death attributed to arrhythmia, heart failure, or unspecified cardiac causes.^7^ CLEAR Outcomes did not make this distinction in its definition of CV death, therefore, we cannot differentiate between coronary and noncoronary CV death with bempedoic acid.

Furthermore, the benefits of bempedoic acid may extend beyond its ability to lower LDL-C. Patients randomized to bempedoic acid had a 21.6% reduction in hsCRP at 6 months, along with a sustained reduction of 17.8% at Month 60. This suggests that there may be additional anti-i nflammatory benefits contributing to the reduction in MACE, although as mentioned above, the CTT analysis suggests the reduction in MACE was consistent with the LDL-C lowering, and argues against any other pleiotropic effects of bempedoic acid driving the outcome reduction. A post-publication analysis of the CLEAR Harmony trial found that in patients with hsCRP ≥2 mg/L, there was no correlation between the ability of bempedoic acid to lower hsCRP and the ability of bempedoic acid to lower LDL-C.^44^ This suggests that its anti-inflammatory properties are independent of its lipid-lowering properties, similar to statins.

The notable side effects of statin therapy, including muscle-related symptoms and new-onset diabetes, have not been found to be associated with bempedoic acid. In the CLEAR Outcomes trial, there was no statistically significant difference in the risk of new-onset diabetes between the two groups. Additionally, the incidence of any muscle-related adverse events and myalgias did not differ between bempedoic acid and placebo (*Figure 3*). It has been suggested that SAMS may be due to the inhibition of HMG-CoA reductase in peripheral tissues. Outside of the liver, HMG-CoA reductase is also used in the production of ubiquinol, a substrate used in cellular energy production, and in post-translational protein prenylation.^45^ The impact of inhibiting HMG-CoA reductase in these cellular processes is thought to contribute to the development of symptoms such as myopathy and hyperglycaemia. Bempedoic acid requires activation by ACSVL1, which is absent in peripheral tissues. This absence of ACSVL1 in peripheral tissues allows for targeted uptake in hepatocytes and may explain the lack of association between bempedoic acid and these adverse effects. However, bempedoic acid has its own unique adverse effect profile, including elevations in uric acid, increased incidence of gout, cholelithiasis and possible tendon rupture; although an increase in tendinopathy was not seen with bempedoic acid in the larger CLEAR Outcomes trial.

Although the results of CLEAR Outcomes are extremely promising, it is important to note the study’s limitations. First, the trial protocol underwent modest protocol changes.^18^ Furthermore, there was a fairly high rate of discontinuation of study drug in both arms (29.1% in the bempedoic acid group and 31.7% in the placebo group); therefore, it may be posited that individuals with statin intolerance may be more likely to be intolerant with taking other medications (i.e. statin intolerance as a marker of suboptimal adherence to medical therapy in general). Nevertheless, despite the discontinuation rate seen in both groups, follow-up in the trial was good, with a primary endpoint available for 95% and vital status for 99% of participants, and analyses were conducted with intention to treat.

In addition, the study protocol allowed for inclusion of patients selfreporting statin intolerance, including those who had only previously attempted therapy with one statin. The National Lipid Association defines statin intolerance as an inability to tolerate a statin dose necessary to achieve patient-specific therapeutic effects, with a minimum of two statins having been attempted.^10^ Statin intolerance can be partial or complete, though complete intolerance is less common. Other professional organizations have similar requirements in their published definitions.^46^ In practice, true statin intolerance according to these definitions is approximately 9.1%.^14^ Therefore, allowing selfreporting of statin intolerance in patients previously attempting therapy with a single statin reduces the number of participants in the trial with true statin intolerance. Furthermore, the study protocol included both primary and secondary prevention patients, and allowed for inclusion of some patients taking low-dose statins, making interpretation of the study results more challenging but again consistent with real-world experience.

Finally, it is important to note that while the Unites States Food and Drug Administration (FDA) mandated that bempedoic acid be studied as a monotherapy in the CLEAR Outcomes trial, in clinical practice it will likely be used in combination with ezetimibe. This combination has been shown to produce an approximate ~40% reduction in LDL-C, and will allow for patients intolerant to statin therapy to achieve similar LDL-C lowering observed with a moderate-i ntensity statin.^33^ Given the success of the CLEAR Outcomes trial, bempedoic acid will be a welcome addition to the lipid-l owering armamentarium of clinicians who care for these patients.

## Considerations for use in contemporary practice

Bempedoic acid originally received FDA approval on 21 February 2020 as an adjunct therapy to diet and maximally tolerated statin therapy for adults with ASCVD or heterozygous FH who require additional LDL-C lowering.^47^ Further, based on the results of the previously referenced phase III trial, bempedoic acid in combination with ezetimibe also received FDA approval in the same patient population on 26 February 2020.^34^ After the successful results of the CLEAR Outcomes trial, it is anticipated that the FDA may approve a label expansion that includes MACE reduction and high-risk primary prevention. At the time of this review article, a request for a change in label has not yet been submitted.

Since the publication of the 2018 American Heart Association/ACC/ Multisociety Blood Cholesterol Guideline, the ACC released its Expert Consensus Decision Pathway (ECDP), which provided updated guidance on the use of non-statin therapies in clinical practice.^5,16^ In addition, the ECDP provided updated LDL-C thresholds for patients with variable levels of absolute risk. There are three main risk groups for whom updated LDL-C thresholds are recommended: patients with clinical ASCVD at very-high-risk for recurrent events, or those with ASCVD and FH should be treated to an LDL-C threshold of <55 mg/dL (non-HDL-C <85 mg/dL); patients with ASCVD not at very-high risk should be treated to an LDL-C threshold of <70 mg/dL (non-HDL-C <100 mg/dL); and patients with baseline LDL-C ≥190 mg/dL without clinical ASCVD should be treated to an LDL-C threshold of <100 mg/dL (non-HDL-C <130 mg/dL). If these thresholds are not achieved with maximally tolerated statin therapy, the addition of non-statin therapies such as ezetimibe or PCSK9 monoclonal antibodies (mAbs) should be considered. If further lowering is needed beyond ezetimibe or PCSK9 mAbs, bempedoic acid or inclisiran should be considered.

The ECDP, which was published in 2022 before the pivotal results of the CLEAR Outcomes trial, also recommends bempedoic acid as second-l ine therapy behind ezetimibe ± PCSK9 mAbs in patients who are unable to achieve their LDL-C thresholds on maximally tolerated statin therapy.^16^ For some patients with statin-associated side effects, their maximally tolerated statin dose may be a low-dose statin or no statin at all. Bempedoic acid, especially in combination with ezetimibe, represents a feasible therapeutic option for patients intolerant to statins requiring significant LDL-C lowering to meet LDL-C thresholds in line with their overall risk. Given its demonstrated ability to provide LDL-C lowering similar to a moderate intensity statin, a bempedoic acid/ezetimibe combination provides patients with statin intolerance with similar LDL-C lowering in an equivalent, simple and once-daily oral dose.^34^

Furthermore, bempedoic acid may be beneficial in patients who are unable to uptitrate their statin medication due to SAMS. In a doseresponse model, adding bempedoic acid to low-dose statin therapy was predicted to achieve similar reductions in LDL-C as quadrupling the dose of statin therapy.^48^ Finally, given the degree of LDL-C lowering and oral dosing, it may be preferable in patients unwilling to take an injectable PCSK9 mAbs or inclisiran.

It is important to note that bempedoic acid should not be considered an alternative to statin therapy. Statins are extremely effective, safe and affordable, and have numerous pleiotropic effects in addition to ASCVD reduction.^49^ In addition, as mentioned previously, the true incidence of statin intolerance is only 9.1%, and a large proportion of SAMS may be due to the nocebo effect.^15^ Many patients are able to tolerate a change in the dosing or frequency of their statin, or a different statin medication. As such, clinicians should continue to attempt to prescribe these incredibly beneficial therapeutics for patients in whom they are appropriate. Lastly, in order to determine whether bempedoic acid is more beneficial than statin therapy, a head-to-head comparison trial would be required. Given the known benefits of statin therapy, this is unlikely to occur as withholding statin treatment from patients able to tolerate them would be unethical.^45^

## Special patient populations

There are specific drug–drug interactions and patient populations in which bempedoic acid warrants further consideration. First, bempedoic acid should not be used in combination with simvastatin >20 mg daily or pravastatin >40 mg daily; however, it can be used with other statins.^22,23^ The use of bempedoic acid at higher doses of simvastatin or pravastatin may increase the concentration of these medications, increasing the risk of statin-associated myopathy.^22,23^ Clinical trials have not studied safety outcomes in patients with estimated glomerular filtration rate <30 mL/min/1.73 m^1^, those with end stage kidney disease on maintenance hemodialysis, or in patients with severe hepatic impairment.

There are no data evaluating the risk of major birth defects, miscarriage or adverse maternal/foetal outcomes. However, based on the mechanism of action of bempedoic acid, the drug may cause foetal harm when given during pregnancy.^22^ As such, it is recommended to discontinue bempedoic acid before pregnancy.

## Conclusion

Despite the proven safety and efficacy of statin therapy, the majority of patients require additional LDL-C lowering in order to meet new guideline-recommended targets to effectively reduce ASCVD risk. Furthermore, statin intolerance leads to a large number of patients in clinical practice who are nonadherent to their guideline-directed lipid-lowering therapy. Bempedoic acid represents a safe, well-tolerated, once-daily, oral medication that specifically targets hepatic cholesterol synthesis and improves CV outcomes, especially in patients with statin intolerance. Furthermore, its use in combination with ezetimibe provides substantially greater LDL-C lowering than either therapy alone. As we continue to target lower LDL-C thresholds in ASCVD prevention, bempedoic acid alone or in combination with ezetimibe is an extremely useful adjunct for primary care clinicians, cardiologists, endocrinologists and lipidologists looking to further reduce LDL-C and improve outcomes for their high-risk patients.
